# Low Cost Three-Dimensional Programmed Mini-Pump Used in PCR

**DOI:** 10.3390/mi13050772

**Published:** 2022-05-14

**Authors:** Chengxiong Lin, Yaocheng Wang, Zhengyu Huang, Yu Guo, Wenming Wu

**Affiliations:** 1 National Engineering Research Center for Healthcare Devices, Guangdong Provincial Key Laboratory of Medical Electronic Instruments and Polymer Material Products, Institute of Biological and Medical Engineering, Guangdong Academy of Sciences, Guangzhou 510316, China; lcx3625@outlook.cn (C.L.); wangyc1126@outlook.com (Y.W.); hzy196521@outlook.com (Z.H.); 2112001219@mail2.gdut.edu.cn (Y.G.); 2 School of Mechanical and Electrical Engineering, Guangdong University of Technology, Guangzhou 510006, China

**Keywords:** mini-pump, PCR, ORF1ab, pipetting accuracy, correction method

## Abstract

Programmed mini-pumps play a significant role in various fields, such as chemistry, biology, and medicine, to transport a measured volume of liquid, especially in the current detection of COVID-19 with PCR. In view of the cost of the current automatic pipetting pump being higher, which is difficult to use in a regular lab, this paper designed and assembled a three-dimensional programmed mini-pump with the common parts and components, such as PLC controller, motor, microinjector, etc. With the weighting calibration before and after pipetting operation, the error of the pipette in 10 μL (0.2%), 2 μL (1.8%), and 1 μL (5.6%) can be obtained. Besides, the contrast test between three-dimensional programmed mini-pump and manual pipette was conducted with the ORF1ab and pGEM-3Zf (+) genes in qPCR. The results proved that the custom-made three-dimensional programmed mini-pump has a stronger reproducibility compared with manual pipette (ORF1ab: 24.06 ± 0.33 vs. 23.50 ± 0.58, *p* = 0.1014; pGEM-3Zf (+): 11.83.06 ± 0.24 vs. 11.50 ± 0.34, *p* = 0.8779). These results can lay the foundation for the functional, fast, and low-cost programmed mini-pump in PCR or other applications for trace measurements.

## 1. Introduction

The outbreak of coronavirus disease 2019 (COVID-19) has killed nearly 5 million people worldwide and infected more than 200 million since 2020, requiring thousands of people to undergo polymerase chain reaction (PCR) detection for the novel coronavirus every day, putting enormous pressure on the healthcare sector [1]. How to improve the efficiency and speed of PCR detection is of great significance for the timely feedback monitoring of the epidemic [2]. However, traditional PCR detection basically requires medical workers to manually complete reagent configuration, which has a series of disadvantages such as high human cost and being time-consuming. Although automated workstations can alleviate the above pressure to some extent, they cannot be widely promoted in remote and backward areas due to the high cost and other problems.

Mini-pumps play a significant role in microfluidic transport, and thus, they are widely used in various fields such as chemistry, biology, and medicine to transport a measured volume of liquid, often as a media dispenser [3]. Mini-pumps come in several designs for various purposes with differing levels of accuracy and precision, from single piece glass pipettes to more complex adjustable or electronic pipettes. Many mini-pump types work by creating a partial vacuum above the liquid-holding chamber and selectively releasing this vacuum to draw up and dispense liquid (e.g., syringe pump [4,5], peristaltic pump [6,7], and piezoelectric mini-pump [8,9]). Although these pumps have prominent advantages, it is high-cost to integrate these pumps into laboratory use or relevant equipment, especially in their fully automatic form.

Programmed mini-pumps are very important in the current rapid-development era, and can save much time during detection or operation, reduce the risk of repetitive strain error from humans, and ensure the reliability of pipetting results with high accuracy and can be used for special occasions, such as radioactive liquid, infectious fluid, or other environments not suitable for human operation [10,11,12]. As a result, with the huge demand of the detection market, more and more attention has been paid to highly automated pipetting systems. For example, as the important detection part of COVID-19, PCR test samples from detecting point need to be divided and mixed in the range of a few microliters to meet the PCR examination requirement [13,14,15,16]. A conventional manual pipette is difficult to meet the sudden outbreak detection and the number of samples tested could reach millions in a medium or large city. On the other hand, it is important to ensure the accuracy on the basis of rapid suction and discharge, especially in small doses [17,18,19].

In view of the high cost of the current pipetting pump, our group independently designed and assembled a three-dimensional programmed mini-pump with the common parts and components, such as programmable logic controller (PLC) controller, motor, microinjector, etc. Through the accuracy test and case comparison in quantitative PCR (qPCR) with manual pipette, it can lay the foundation for the functional, fast, and low-cost programmed mini-pump in PCR or other applications for trace measurements.

## 2. Materials and Methods

### 2.1. Basic Composition

**Figure 1:**
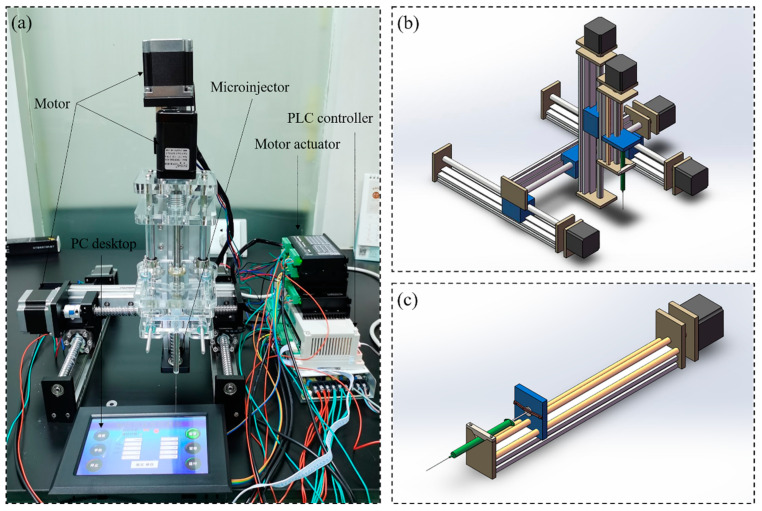
The three-dimensional precision automatic pipette: (**a**) picture of real products, (**b**) schematic diagram for total equipment, (**c**) schematic diagram for mini-pump.

The custom-made three-dimensional programmed mini-pump was mainly made of PC desktop (Lenovo, Shenzhen, China), PLC controller (Shenzhen Bianneng technology, Shenzhen, China), motor actuator (CNYOHO, Wenzhou, China), motor (CNYOHO, Wenzhou, China) for X/Y/Z axis and microinjector (Gaoge, Shanghai, China), and corresponding guide rail (Fanggong, Taizhou, China), as shown in Figure 1. The movement in X/Y/Z direction of the equipment is mainly driven by the screw rod connected the motor and screw rod drive slider. The guide rail in different directions was mounted on the slider and the pipette was mounted on the slider in Z direction. As the core component, the slider near the microinjector was replaced with a baffle for advancing or withdrawing the end of the microinjector. In the current experiment, the measurement range of the microinjector was 10 μL. Before the experiments, program commands could be entered into the PLC controller from the PC desktop (there is a typical program command in the Appendix A as an example). The overall principle is that the suction or drainage of the microinjector occurred after the positions in three directions were determined.

### 2.2. Accuracy Verification

**Figure 2:**
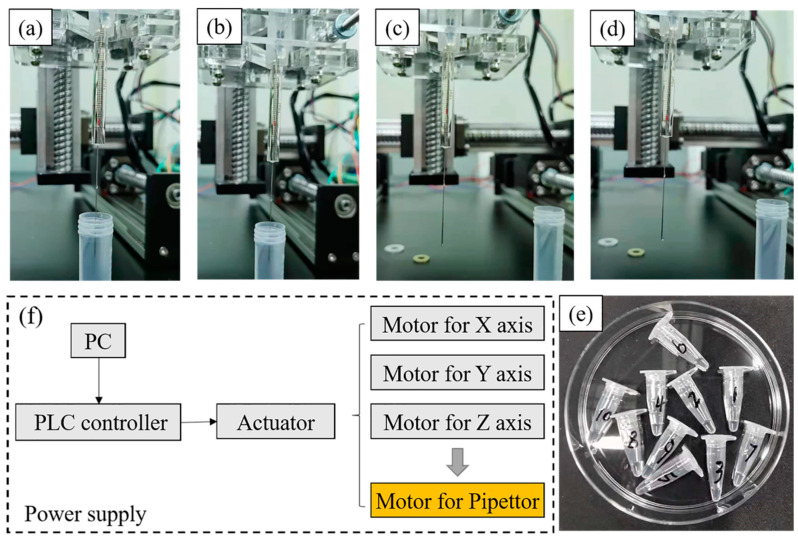
The process of suction and discharge from (**a**–**d**), (**e**) is the Eppendorf tubes used for collecting liquid, (**f**) is the basic principle of the equipment.

In order to verify the pipetting accuracy of the above custom-made three-dimensional precision automatic pipette, the relative verification experiment was carried out according to the weighing method [20]. Through measuring the weight of numbered containers before and after the experiment with a standard balance (precision 0.1 mg), the volume of the liquid can be obtained from the density (Figure 2). In the current experiment, the liquid used for accuracy verification was deionized water (1 g/mL in 4 °C) and there were three volume standards (10 μL, 2 μL, 1 μL). For the manual pipetting experiment of 10 μL, the total of liquid was 50 μL in a continuous pipette, namely 5 times, and the latter two was 20 μL (10 times for 2 μL and 20 times for 1 μL). All the accuracy verification experiments were repeated 10 times to avoid error. Besides, the comparison object was a commercially available equivalent system in order to reduce the cost. The related accuracy-defined formula is listed below:

(1) $$Error=\frac{V_{measured}-V_{designed}}{V_{designed}}$$

where $V_{measured}$ and $V_{designed}$ both mean the single operation.

### 2.3. PCR Verification

In addition, to prove the three-dimensional precision automatic pipette which can be used in laboratories, fluorescence experiments were conducted in a commercial real-time qPCR (Connect, Bio Rad, Hercules, CA, USA) with a manual pipette (Pipet-Lite, Rainin, Oakland, CA, USA) as reference. By comparing the fluorescence intensity and the product of the two devices, the performance of the microdevice could be verified. PCR Reagents contained a buffer composed of 10 μL premix, 1 μL forward primer, 1 μL reverse primers, 1 μL eva green, 1 μL DNA template, and 6 μL DI water. There were two primer sequences were listed in Table 1:

The gene of ORF1ab and pGEM-3Zf (+) was inserted into pUC57 plasmid vector (Sangon Biotech, Shanghai, China) by recombinase, which was further used as the PCR target. Fluorinated oil (HFE 7500, 3M Novec, San Diego, CA, USA) was used as the oil phase, and agarose powder (V900510, Sigma-Aldrich, MO, USA) was used for agarose gel electrophoresis. Besides, the concentrations of the DNA template were changed (10^4^, 10^5^, 10^6^, 10^7^ copies/µL). Before the Eppendorf tubes were moved to the qPCR, centrifugal operation was conducted to mix up the above 6 liquids. The two-steps cyclic heating process in qPCR was 95 °C for 15 s and 60 °C for 35 s. The heating cycle was 40 times.

### 2.4. Statistics

In the tests of accuracy verification, the experimental data were presented by the mean and standard deviation. F test (analysis of variance) was used to determine the significant difference among different esophageal samples under the same test conditions. The level of statistical significance was set to *p* < 0.05.

## 3. Results and Discussion

### 3.1. Verification of Pipetting Accuracy by Weight Methods

**Figure 3:**
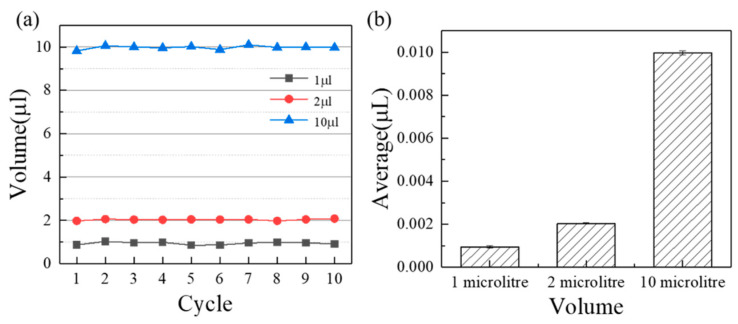
The measurement results of the three-dimensional precision automatic pipette in 10 μL, 2 μL, 1 μL: (**a**) Overall results, (**b**) Average results.

According to the measuring principle in Section 2.2, the accuracy can be calculated as shown in Figure 3. The average error volume of suction and discharge are $2.0\times 10^{-5\;}L$, $3.6\times 10^{-5}\;L,$ and $5.6\times 10^{-5}\;L$, namely, the corresponding accuracies are 0.2% (10 μL), 1.8% (2 μL), and 5.6% (1 μL). It can be found that the pipetting accuracy increases with the increasing measuring volume for a single injector (*p* < 0.05). On the one hand, for an air displacement pump, the smaller the volume, the more difficult its precision control is. On the other hand, as the volume decreases, the emptying of the injection pump becomes more difficult due to surface tension of DI water. In a word, sealing is a guarantee of accuracy of the pump and the influence of surface tension aggravate when the volume reduces to a certain degree. As a contrast, current publications paid more attention to the liquid volume range around 10 μL for microfluidics, such as peristaltic pump [21] and pressure pump [22]. The current accuracy of commercial pipetting pumps at 1 μL, 10 μL are 5%, 1.5% respectively (https://craft-oem.s3.amazonaws.com/File-Uploads/695216-01_ZeusLT_SpecSheet_A4_EN_LR.PDF?v=1,557,757,671, accessed on 6 May 2022), such as ZEUS and ZEUS^@^LT (Hamilton, Switzerland), S1 pipetting workstation (Superh2, Foshan, China). As a result, the custom-made three-dimensional programmed mini-pump has a comparable pipetting accuracy. Furthermore, the expense of the custom-made three-dimensional programmed mini-pump, namely the assembly price, reduces to a large extent (250 US Dollar) under the condition of meeting basic pipetting accuracy, which was much less than the commercial equipment (4000 US Dollar for ZEUS or ZEUS^@^LT).

### 3.2. Typical Application in Virus Detection with qPCR

**Figure 4:**
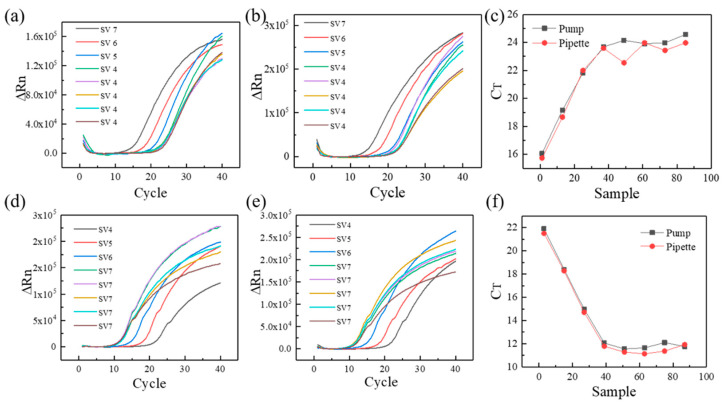
The qPCR results of different template DNA concentration: (**a**) relative fluorescence intensity of the custom-made three-dimensional automatic pipette for ORF1ab, (**b**) relative fluorescence intensity of the manual pipette for ORF1ab, (**c**) initial template DNA concentration for ORF1ab, (**d**) relative fluorescence intensity of the custom-made three-dimensional automatic pipette for pGEM-3Zf (+), (**e**) relative fluorescence intensity of the manual pipette for pGEM-3Zf (+), (**f**) initial template DNA concentration for pGEM-3Zf (+). The *x*-axis in (**c**,**f**) means different samples (SV 4, SV 5, SV 6, SV 7).

As the typical detected gene in COVID-19, ORF1ab was selected as the feature gene in the current experiment [23,24]. As shown in Figure 4a,b, SV4, SV5, SV6, and SV7 represent the different template DNA concentration (10^4^, 10^5^, 10^6^, 10^7^ copies/μL, respectively). The 5 repetitions of samples with a template DNA concentration of 10^4^ copies/µL were tested for consistency and stability of PCR results, and the samples with gradually varied concentration can verify the correctness of suction or drainage. The fluorescence intensity of different reaction mixture increases gradually as the reaction proceeds. Compared to the manual pipette, the relative fluorescence intensity of the custom-made three-dimensional programmed mini-pump presents good coincidence performance at the template DNA concentration of copies/µL. Meanwhile, the differences are also obvious for different template DNA concentration. As can be seen from Figure 4c, the same template CT value has a stronger reproducibility for the custom-made three-dimensional programmed mini-pump compared with manual pipette (24.06 ± 0.33 vs. 23.50 ± 0.58, *p* = 0.1014), which shows a manual pipetting error for second sample with a concentration of 10^4^ copies/µL. For current laboratory, manual pipettes are the main pipetting tools with a range of 1–1000 μL. As a result, the custom-made three-dimensional programmed mini-pump can become one of pipetting options in an automatic mode. Besides, the similar fluorescent results of pGEM-3Zf (+) were shown in Figure 4d–f, and the template CT value shows similarities for the custom-made three-dimensional programmed mini-pump and manual pipette (11.83.06 ± 0.24 vs. 11.50 ± 0.34, *p* = 0.8779).

## 4. Conclusions

As a pipetting system, this paper describes a custom-made three-dimensional programmed mini-pump consisting of common PC desktop, PLC controller, motor actuator, motor for X\Y\Z axis, and pipettor and corresponding guide rail. Through a standard correction method for accuracy and PCR verification for application, the three-dimensional precision automatic pipette was verified. The conclusions can be summarized in two main parts:

1. A custom-made three-dimensional programmed mini-pump was assembled with an accuracy of 0.2% for 10 μL, 1.8% for 2 μL, and 5.6% for 1 μL.
2. Relative to manual pipette, three-dimensional programmed mini-pump has a stronger reproducibility in PCR application (ORF1ab: 24.06 ± 0.33 vs. 23.50 ± 0.58, *p* = 0.1014; pGEM-3Zf (+): 11.83.06 ± 0.24 vs. 11.50 ± 0.34, *p* = 0.8779).
3. The current programmed mini-pumpscan be realized with low cost.

## Figures and Tables

**Table 1:** Primer sequences in PCR verification experiment.

	ORF1ab	pGEM-3Zf (+)
Forward	5′ CCCTGTGGGTTTTACACTTAA 3′	5′ GGAGAGGCGGTTTGCGTATTGGG 3′
Reverse	5′ ACGATTGTGCATCAGCTGA 3′	5′ TTTGTGATGCTCGTCAGGGGGGC 3′

## Appendix A. Supplementary Data

**Table A1:** The typical program command for the three-dimensional automatic pipette.

Program Command=
>001: N1;
>002: Z-30;
>003: F2000;
>004: A-@1;
>005: F1200;
>006: L2000;
>007: Z+30;
>008:X+60;
>009: Z-30;
>010: F2000;
>011: A+@1;
>012: F1200;
>013: L2000;
>014: Z+30;
>015: X-60;
>016: N2=@2;
>017: END;

Note: @ can be used for an External adjustable window for different volume of pipetting and cycle index.
